# Watermelon fruit metabolome gene discovery and its application in breeding: a review

**DOI:** 10.3389/fpls.2025.1687406

**Published:** 2025-10-17

**Authors:** Fikru Tamiru Kenea, Nan He, Xuqiang Lu, Xiaowen Luo, Hongju Zhu, Wenge Liu

**Affiliations:** ^1^ Henan Joint International Research Laboratory of South Asian Fruits and Cucurbits, Zhengzhou Fruit Research Institute, Chinese Academy of Agricultural Sciences, Zhengzhou, China; ^2^ Department of Horticulture, College of Agricultural and Natural Resources, Dilla University, Dilla, Ethiopia; ^3^ Zhongyuan Research Center, Chinese Academy of Agricultural Sciences, Xinxiang, China

**Keywords:** citrullus lanatus, metabolites, gene discovery, genomics, marker-assisted selection, fruit quality

## Abstract

Watermelon [*Citrullus lanatus* (Thunb.) Matsum. & Nakai] is a globally important vegetable crop valued for its taste, hydration, and nutritional benefits. Recent advances in multi-omics technologies have accelerated the identification of genes controlling key fruit metabolites that impact fruit quality traits such as sweetness, bitterness, sourness, aroma, texture, and color. This review synthesizes the current knowledge on watermelon genes regulating and transporting fruit metabolites, including sugars, cucurbitacin, organic acids, carotenoids, amino acids, flavonoids, and volatile organic compounds that impact fruit quality. Both forward and reverse genetics approaches, coupled with high-throughput phenotyping, have been instrumental in these gene discoveries. Breeding applications, including marker-assisted selection (MAS) and genomic selection (GS), are highlighted, emphasizing their potential to enhance fruit metabolites that improve fruit quality and nutritional value. Emerging technologies, such as CRISPR/Cas9-mediated gene editing, have been employed to uncover and validate *CIVST1*, *PSY1*, and *PEPCK* genes, enabling precision breeding for improved fruit metabolite profile. However, challenges persist due to the environmental sensitivity and polygenic nature of fruit metabolites, the narrow genetic base, and the limited adoption of molecular breeding methods like CRISPR/Cas9. Future directions emphasize leveraging wild germplasm, integrating AI-driven phenotyping, and applying precision breeding strategies. These approaches will enable the development of next-generation watermelon cultivars with improved multi-trait quality and nutritional profiles to meet evolving market demands.

## 1 Introduction

Watermelon (*Citrullus lanatus*) is a globally significant fruit crop known for its refreshing taste, high water content, and nutritional value (Sorokina et al., 2021; Dubey et al., 2023). As consumer demands evolve, there is increasing interest in developing watermelon cultivars with superior fruit quality traits, including sweetness, color, aroma, and nutritional content (Assefa et al., 2020; Ramirez et al., 2020). A key focus for maintaining this feature is examining the fruit metabolome, which encompasses all small molecules influencing the flavor and nutritional characteristics of the fruit. Though it is a time-consuming, expensive, and distractive approach, recent liquid chromatography–mass spectrometry (LC-MS/MS), ultra-high-performance liquid chromatography, gas chromatography-mass spectrometry (GC-MS), and nuclear magnetic resonance (NMR) spectroscopy have revolutionized watermelon fruit metabolome investigation. Furthermore, the cutting-edge and powerful non-destructive technique, Raman spectroscopy, possibly enhanced with artificial intelligence (AI), has been used to confirm the presence and intensity of carotenoids, particularly lutein and β-carotene, in watermelon fruit (Dhanani et al., 2022). Surprisingly, gradual adoption of generative AI, particularly by ChatGPT, compared with human perception for internal quality based on outer characteristics of watermelon fruit, has revealed significant acceptance of internal fruit quality expected by consumers (Ozdemir, 2025). Though it has drawbacks, exploring this breakthrough technology for other traits and genetic makeup will enable scientists to investigate thousands of fruits for desirable metabolomes and associated molecular mechanisms within a short period, at low cost, and without distracting the fruit.

The watermelon fruit metabolome consists of a diverse array of primary and secondary metabolites that influence taste, texture, appearance, and nutritional quality (Zhang and Hao, 2020; Gong et al., 2021b; Guo et al., 2015; Chu et al., 2022; Ning et al., 2024; Peng et al., 2025). Primary metabolites such as sugars and organic acids directly impact sweetness and sourness (Guo et al., 2019, Guo et al., 2015; Umer et al., 2020a, Umer et al, 2020b), while secondary metabolites like carotenoids (Guo et al., 2019; Ilahy et al., 2019; Sulaiman et al., 2020; Breniere et al., 2022; Lv et al., 2022; Peng et al., 2025) and volatiles affect color and aroma (Kyriacou et al., 2018; Bianchi et al., 2019; Gong et al., 2023, Gong et al., 2021a; Liu et al., 2018; Tripodi et al., 2019). Genetic and environmental factors tightly regulate the dynamic composition of these metabolites during fruit development (Soteriou and Kyriacou, 2015; Liu et al., 2018; Aslam et al., 2020; Trandel et al., 2021). Yuan et al. (2021a) has identified more than 431 metabolites, while Sorokina et al. (2021) reported 1,679 with different contents. This reveals that metabolite contents demand further investigation to exploit their potential among different germplasm and their causal genes.

Improving watermelon cultivars through conventional approaches has not only been time consuming but has also resulted in the inheritance of undesirable traits along with desired metabolites. Since the conventional breeding approaches have not been able to meet the demand arising from different dimensions, uncovering the genes of different metabolites has emerged to advance molecular breeding in watermelon fruit. Advances in high-throughput like multi-omics technologies have facilitated the identification of genes involved in the biosynthesis, degradation, and transportation of these metabolites (Kim and Buell, 2015; Gong et al., 2021b; Guo et al., 2015; Ren et al., 2021). This knowledge has been integrated into modern breeding programs through marker-assisted selection (MAS), genomic selection (GS), gene transformation, and gene editing, ultimately enabling the development of high-quality watermelon cultivars tailored to specific market and health demands (Fall et al., 2019; Guo et al., 2019; Mashilo et al., 2022; McGregor et al., 2023; Verma et al., 2024). The whole genome sequencing of watermelon, the “97103” elite cultivar (Guo et al., 2012) and its improved sequence (Guo et al., 2019), the “Charleston Gray” cultivar (Wu et al., 2019), the closest relative of the cultivated watermelon, the “Kordofan melon” (Renner et al., 2021), and the telomer-to-telomer gap-free genome of the elite watermelon inbred line “G42” (Deng et al., 2022) have all been used as a reference for watermelon gene discovery and genetic improvement. Furthermore, comparative analysis of 20 (Guo et al., 2012) and 414 resequenced germplasm (Guo et al., 2019b) and genomes of 1,365 accessions (Wu et al., 2019), as well as the super-pangenome from 547 different accessions, has been used to uncover candidate genes of various metabolites (Wu et al., 2023). For instance, the integration of metabolomics and genomics has identified the sugar transporter gene ClTST2 (Cla97C02G036390) and the synthase gene Cla97C10G194010 that have been harbored on chromosome 10 and associated with fruit sweetness (Guo et al., 2019). Similarly, the lycopene β-cyclase gene (LCYB, Cla97C04G070940) that manipulates the flesh color has been identified on Chromosome 4 (Liu et al., 2016; Guo et al., 2019).

By integrating metabolomics data with genomic tools such as quantitative trait locus (QTL) mapping and genome-wide association studies (GWAS), researchers have pinpointed specific genes responsible for desirable metabolic traits (Wang et al., 2021a; Liang et al., 2022; Zhan et al., 2023). Furthermore, these approaches have been used to reveal regulatory networks (Ren et al., 2014; Katuuramu et al., 2023; Vallarino et al., 2023). This systems-level understanding enables breeders to target metabolic pathways more effectively in the selection process. Similarly, the integration of other omics, such as transcriptomics and proteomics, with metabolomics has revolutionized gene discovery (Abdullah-Zawawi et al., 2022; Depuydt et al., 2022; Habibi et al., 2024; Shiratake and Suzuki, 2016). Transcriptomics provides insights into gene expression patterns during fruit development and ripening, while metabolomics quantifies the levels of various metabolites (Umer et al., 2020b; Gong et al., 2021a). The correlation between gene expression and quantified metabolite level has identified candidate genes that have been either upregulated or downregulated to alter the metabolite accumulation. For instance, upregulation of Cla97C03G064990 (sucrose synthase) and Cla97C03G068240 (citrate synthase) has been highly correlated with the accumulation of sucrose and citric acid, respectively, in watermelon fruit flesh (Umer et al., 2020b; Peng et al., 2025). Cla018406 (a chaperone protein, DNAJ-like protein) and Cla007686 (a zinc finger CCCH domain-containing protein) transcripts have been highly expressed in yellow- and red-fleshed watermelon, revealing high β-carotene and lycopene accumulation (Wang et al., 2021a; Yuan et al., 2021b). Systems biology approaches further enhance the understanding of the complex interactions between genes and metabolites, paving the way for more targeted breeding strategies. The use of multi-omics data thus represents a powerful approach for unraveling the molecular basis of the fruit metabolome.

The current review attempts to summarize and provide critical insights into recent watermelon metabolome gene discoveries that regulate key metabolic pathways. It aims to highlight recent advances in forward and reverse metabolomics-assisted uncovered genes and explore how cutting-edge tools, including MAS, GS, and gene editing, can be utilized to develop watermelon cultivars with a superior fruit metabolome. By addressing challenges such as limited resources, a shortage of skilled human power, insufficient investigation of multi-omics and AI integration, environmental sensitivity, and limited genetic diversity, this review also discloses the transformative potential of modern breeding strategies in enhancing the watermelon fruit metabolome.

## 2 Gene discovery approaches

To characterize the inherent and enormously diversified resources of plants for better management and efficient utilization in crop improvement, proper phenotyping and genotyping of a large set of plant genetic resources is crucial. Rapid and precise phenotypic assessment of thousands of breeding lines, clones, or populations over time under diverse environments is the only critical component for accelerating the development of new and improved cultivars through discovering elite genes (Fu, 2015; Sun et al., 2024). The plant phenotype refers to all morphological, physiological, and biochemical characteristics, which are controlled by genotype and environment, reflecting the structure, composition, and growth of a plant to identify key genes (Fiorani and Schurr, 2013; Johannsen, 2014; Pontarotti et al., 2022). This is satisfied through acquisition of high-dimensional phenotypic data [high-throughput phenotyping (HTP)] (Chawade et al., 2019; Kim, 2020) or “phenomics” (Houle et al., 2010; Zhao et al., 2019), technologies that help for the discovery of important genes. Complex characters that are relevant for plant selection (forward phenomics) and explanations of why the given genotypes stand out in a specific environment (reverse phenomics) are predicted from this method. Identifying genetic architectures through linking phenotypes and genotypes is used to regulate important traits that are used for plant breeding and development of plant genomics (Xiao et al., 2021).

**Figure 1:**
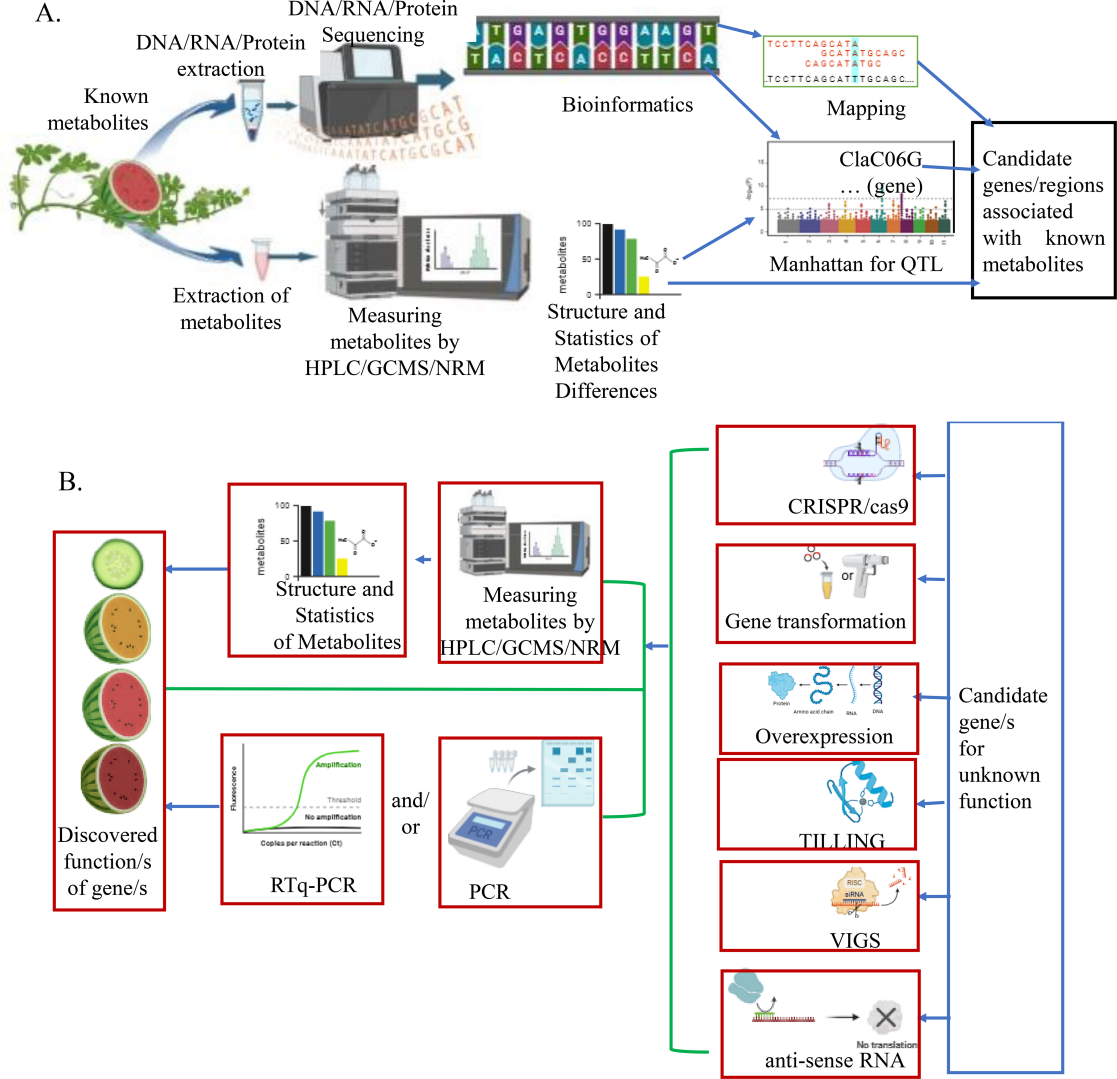
Examples of a short summary of forward and reverse genetics: **(A)** Forward gene discovery. **(B)** Reverse gene discovery (these figures were illustrated by biorender, an online tool).

Gene discovery can be done through forward genetics (phenotype to gene) and reverse genetics (gene to phenotype) approaches (**Figure 1**). Forward genetics (traditional), the phenotype gene approach, is an approach used to find the genetic basis of a particular phenotype of an organism (Brown et al., 2005). It identifies and characterizes genes (or sets of genes) responsible for mutant phenotypes or traits. In this approach, the phenotypic character of the plant is evaluated, and a novel plant genotype is selected (Jankowicz-Cieslak and Till, 2015). A plant with desired character is incorporated into breeding programs and/or used for further understanding of its genetic mechanisms and function validation. After the reference genome of watermelon was deciphered, several forward gene discovery methods, including genome-wide association analysis (GWAS), bulk segregant analysis, QTL mapping, and transcriptomics, were employed to uncover desirable genes for intended metabolites.

Reverse genetics, which is known as gene to phenotype, is based on known gene sequences to find particular phenotypes (functions of genes) (Bouché and Bouchez, 2001). It involves finding the result of particular genetic sequences induced by a mutagen. This strategy works on the single gene which its function or the phenotype it creates is not known (Brown et al., 2005).

The first step for both forward and reverse genetics is obtaining a suitable mutagenized population through natural or induced mutagenesis (Serrat et al., 2014). The steps of forward genetics are getting a mutant which is a naturally varied or induced mutation, followed by selecting a phenotype to identify the gene and its function (Zakhrabekova et al., 2013). These authors revealed that the efficiency is increased through emerging technology like genetic mapping, quantitative trait loci mapping, and the exome sequencing approach. In the case of reverse genetics, the known sequence of a gene, whose function is unknown, is induced or altered in expression and followed by discovering the function of the gene by analyzing its phenotypic effects. Different tools and techniques used for reverse genetic approaches are genetic engineering, gene silencing, zinc finger nuclease, Targeting Induced Local Lesions in Genomes (TILLING), CRISPR/Cas9, T-DNA, transposons, mutagenesis, overexpression, and homologous chromosomes (Jankowicz-Cieslak and Till, 2015).

To manage diversity and prove effective in increasing long-term genetic gain, methodologies like genetic marker-based information have been developed (Jannink et al., 2010). It is important for developments in the field of plant breeding (Kebriyaee et al., 2012). It is a gene or DNA sequence with a known chromosome location controlling a particular gene or trait. Genetic markers act as signs or flags and closely related to the targeted genes (Collard et al., 2005). Genetic markers are broadly grouped into classical markers (morphological, cytological, and biochemical) and DNA/molecular markers. But some literature classifies it as a morphological and molecular (biochemical and DNA) marker. Restriction fragment length polymorphism (RFLP), amplified fragment length polymorphism (AFLP), simple sequence repeats (SSRs), single nucleotide polymorphism (SNP), and diversity arrays technology (DArT) markers are some examples of molecular markers (Wenzl et al., 2006). Pyramiding all levels of information (different categories of traits measured at different times with environmental information) improves crop germplasm.

## 3 Watermelon fruit metabolome and their genes

### 3.1 Sugar-related genes

**Figure 2:**
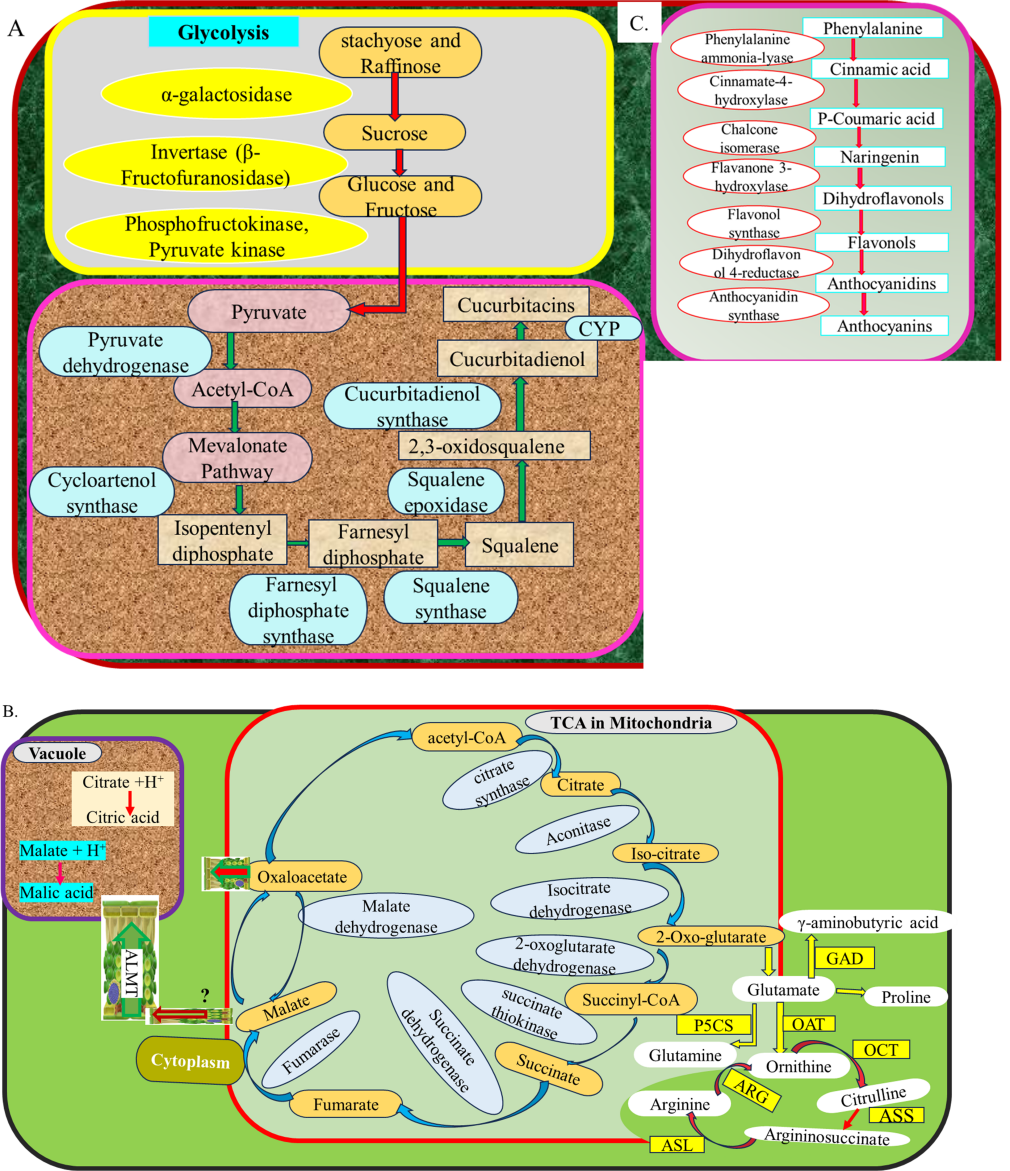
Genes involve in different metabolic networks in watermelon fruits **(A)** sugar and cucurbitacin biosynthesis **(B)** Organic acid and amino acid biosyntheis **(C)** Flavonoids biosynthesis.

Sweetness is one of the most desirable traits in watermelon and is primarily determined by the accumulation of soluble sugars, such as sucrose, glucose, and fructose (Guo et al., 2019, Guo et al., 2015; Umer et al., 2020a, Umer et al., 2020b). Key genes involved in sugar metabolism include sucrose phosphate synthase (SPS), sucrose synthase (SUS), and various invertases (INVs), which regulate sucrose synthesis and degradation (Guo et al., 2019; Umer et al., 2020a, Umer et al., 2020b) (**Table 1**). Sweet watermelon varieties have exhibited higher unloading of raffinose and stachyose compared to non-sweet varieties, which has been correlated with increased sugar content (Liu et al., 2013). Gao et al. (2018b) reported that the cultivar “203Z” has produced higher sugar content than “SW,” while the higher acid content has been produced by “SW.” Different varieties of sweet watermelon have produced different levels of soluble sugar and enzymes that have been involved during their metabolism (Yativ et al., 2010). The sugar content of sweet watermelon is increased due to hydrolysis of raffinose and stachyose by galactosidases and is transported by ClVST1, ClSWEET3, and ClTST2 genes, which have been selected during evolution and domestication (Ren et al., 2021). Specific genes, such as ClAGA2, essential for the hydrolysis of oligosaccharides and sugar transport, influence the partitioning of carbohydrates in sweet watermelon fruits (Ren et al., 2021). For instance, nine α-galactosidase genes have been identified in the watermelon genome, and the insoluble acid invertase (IAI) gene (Cla020872) has been highly expressed in the mesocarp of elite 97103 than in PI296341-FR fruit flesh, resulting in high sugar accumulation in 97103 (Guo et al., 2015). Similarly, raffinose and stachyose, the major translocated sugars, have been less unloaded into non-sweet line PI296341-FR (*C. lanatus* subsp. *lanatus*) than elite sweet watermelon line 97103 (*Citrullus lanatus* subsp. *vulgaris*) fruit (Liu et al., 2013). On the other hand, insoluble acid invertase, the Cla020872 gene, which has been strongly correlated with enzyme activity in the flesh and the mesocarp of fruit, has been upregulated in 97103 but is at much lower levels in the flesh of PI296341-FR (Guo et al., 2015; Liu et al., 2013). This has resulted in fructose and glucose translocation and the intercellular sugar accumulation in 97103 watermelon fruit through extracellular sucrose degeneration (Guo et al., 2015). Some of the genes involved in the simple sugar formation pathway are illustrated in the following **Figure 2A**.

**Table 1:** Uncovered genes of sugars and cucurbitacin of watermelon fruits.

Traits	Gene/QTL	References
Sugar	ClVST1 (Cla97C02G031010), ClAGA2 (Cla97C04G070460), ClSWEET3 (Cla97C01G000640), and ClTST2 (Cla97C02G03639)	(Gong et al., 2021b)
	Cla000264 (Tonoplast Sugar Transporter)	(Sun et al., 2020)
	ClTST2 (Tonoplast Sugar Transporter)	(Ren et al., 2021)
	ClSWEET3 (Sugars Will Eventually Be Exported Transporter 3)	
Raffinose	Raffinose synthase (Cla97C10G196740)	(Guo et al., 2019)
Raffinose and Stachyose	ClAGA2 (alkaline alpha-galactosidase) Cla013902	(Ren et al., 2021)
Trehalose	trehalose-6-phosphate synthase (Cla97C10G186050) and trehalose 6-phosphate phosphatase (Cla97C08G153950)	(Gong et al., 2021b)
	Cla011768 (Membrane transporter)	(Pingli et al., 2023)
Sucrose	SuSy (Cla97C10G194010) and WMU23179 (SPS) (Cla97C06G117750, Cla97C11G215270)	(Guo et al., 2011; Gong et al., 2021b)
	Qsur2–1 and Qsur2-2, Qsur5	(Ren et al., 2014)
Fructose	fructose-bisphosphate aldolase (Cla97C10G202380, Cla97C09G183030, Cla97C04G076620, Cla97C01G006270)	(Gong et al., 2021b)
	Cla97C01G003970 (β-fructofuranosidase) (VCINV)	(Peng et al., 2025)
	Cla97C05G094630 (Fructokinase)	(Umer et al., 2020a)
	Cla97C04G076620 (Fructose bisphosphate aldolase)	
	Cla97C08G147060 (Fructose 1-6, bisphosphate)	
	pyruvate kinase gene (Cla97C05G090710 and Cla97C06G116090]	(Peng et al., 2025)
	Qfru2-1, Qfru2–2 and Qfru2-3	(Ren et al., 2014)
	Qfru6, Qfru8	
Glucose	glucose-6-phosphate isomerase (Cla97C08G154900, Cla97C08G158730)	(Gong et al., 2021b)
	phosphoglucomutase (Cla97C01G006680)	
	hexokinase (Cla97C02G049120)	(Peng et al., 2025)
	Cla97C11G223580 (PEPCK)	(Umer et al., 2020a)
	Qfru6	(Ren et al., 2014)
Cucurbitacin	Cla007080 (Cycloartenol synthase)	(Chai and Sun, 2025)
	Cla97C01G011050 (Farnesyl diphosphate synthase)	
	Cla97C11G210690 (Squalene synthase)	
	Squalene epoxidase(WMU52805, WMU57355)	
	Cycloartenol synthase (Cla007080)	
	UDP-glycosyl transferase (Cla97C09G164970, Cla97C02G045300, Cla97C03G051170, Cla97C03G051970, Cla97C03G051950, Cla97C09G174190, Cla97C09G174200)	(Gong et al., 2021b; Chai and Sun, 2025)
	Cla011464 (Glycosyl transferase)	(Pingli et al., 2023)
	Cla011487 (ERF), bHLH (Cla011508, Cla004098 and Cla011510), Cytochrome P450 (Cla011514 and Cla011515), Ds synthase (Cla004749 and Cla019330)	
	Cytochrome P450s (CYP)	(Zhou et al., 2016; Chai and Sun, 2025)
	Acyltransferase (ACT)	
	Oxidosqualene cyclase (OSC)	(Zhou et al., 2016)

The gene expression studies of specific genes, such as Cla97C01G000640 and Cla97C05G087120, which have been identified as sugar transporters, have been strongly correlated with sugar content (Umer et al., 2020a). The NAC transcription factor ClNAC68 has repressed the ClINV and ClGH3.6 genes and positively regulated sugar, thereby enhancing fruit sweetness (Wang et al., 2021b).

Genetic mapping studies have also identified major quantitative trait loci (QTLs) linked to sugar content on chromosome 10 (Guo et al., 2019). These findings will enable the development of molecular markers, allowing breeders to select high-sugar genotypes early in the breeding process, thereby significantly improving breeding efficiency. Moreover, unlocking these genes is the backbone of gene editing and mutant development, which requires more emphasis.

### 3.2 Cucurbitacin-related genes

Cucurbitacins, bitter-tasting compounds prevalent in wild watermelons, are mostly absent in cultivated varieties (Zhou et al., 2016). Deciphering the genes responsible for cucurbitacin biosynthesis and regulation is crucial for breeding efforts and enhancing fruit quality. Recent studies have uncovered critical genes and regulatory mechanisms that govern cucurbitacin production in watermelon fruit. Cucurbitacin biosynthesis in watermelon has been regulated by a coordinated network of structural genes, modifying enzymes, and transcriptional regulators. Key genes, such as the *CGC1* cluster, cytochrome P450s, cucurbitadienol synthase, and various transferases, play central roles in the production and diversification of cucurbitacins (Zhou et al., 2016; Chai and Sun, 2025) as illustrated in **Figure 2C**. It was indicated from pyruvate to cucurbitacin in this figure. Fine mapping of F_2_ population of watermelon that has been developed from two inbred lines, “9904” (bitter flesh) and “Handel” (non-bitter flesh), with the parents, has enabled the uncovering of the locus of four candidate genes (*Cla011507*, *Cla011508*, *Cla011509*, and *Cla011510*) that synthesize watermelon fruit cucurbitacin (Gong et al., 2022). Pingli et al. (2023) have carried out genome-wide analysis and reported 42 watermelon fruit cucurbitacins with 8 major loci. Especially cytochrome P450s genes create diversity of cucurbitacins (Zhou et al., 2016). Through a biosynthesis of cucurbitacins, cucurbitadienol, which is the triterpenoid, has been utilized as a substrate for the production of cucurbitacins. It has been synthesized from 2,3-oxidosqualene by an oxidosqualene cyclase gene (Davidovich-Rikanati et al., 2015). Transcription factors of oxidosqualene cyclase (OSC), ACT, and CYP genes have also demonstrated high production of cucurbitacin in wild watermelon, while it has not been revealed in domesticated watermelon (Zhou et al., 2016). Similarly, SNP change in the transcription factor binding site (GCC-box) in promoter regions of cultivated watermelon has banned the production of cucurbitacins (Pingli et al., 2023). Furthermore, these authors reported the insertion of 244 amino acids in the CDS region of Cla019330 (ClOSC) in cultivated watermelon, while that insertion has been absent in wild watermelon and resulted in the absence of cucurbitacin production. This is deciphered as domestication has led to reduced expression and accumulation of these bitter compounds. The other reported genes have been revealed in the following **Table 1**. These genes need further validation and implementation for molecular breeding.

### 3.3 Carotenoid-related genes

Watermelon carotenoids are known for their antioxidant properties and potential health benefits (Edwards et al., 2003). Moreover, the flesh color of watermelon, a key quality attribute, has been determined by the presence of carotenoid pigments such as lycopene, β-carotene, violaxanthin, and lutein (Breniere et al., 2022; Ilahy et al., 2019; Lv et al., 2022; Peng et al., 2025; Sulaiman et al., 2020). Yuan et al (2021a) reported candidate genes of β-carotene, lycopene, and xanthophyll (such as violaxanthin and lutein) accumulation, which have been regulated by Cla018406 (a chaperone protein DNAJ-like protein), Cla007686 (a zinc finger CCCH domain-containing protein), and Cla021635 (photosystem I reaction center subunit II), have controlled the formation of orange, red, and yellow flesh-colored watermelon fruit, respectively. These color variations have been genetically regulated by key enzymes in the carotenoid pathway, including phytoene synthase (PSY1), lycopene β-cyclase (LCYB), and β-carotene hydroxylase (CHYB) (Nie et al., 2023) (**Table 2**).

**Table 2:** Uncovered major genes of carotenoids, organic acids, and texture-related traits of watermelon fruits.

Traits	Gene/QTL	References
Citric acid	citrate synthase (Cla97C03G068240, Cla97C08G161610)	(Gong et al., 2021b)
	Cla97C03G068240 (citrate synthase)	(Umer et al., 2020a)
	Cla97C01G008870 (isocitrate dehydrogenase)	
	Cla013500 (citrate synthases)	(Gao et al., 2018a)
	WMCS	(Umer et al., 2020b)
	Cla007935 (Auxin efflux carrier)	(Pingli et al., 2023)
	cit7-10, cit9-32, cit10-16, cit11-9, and cit11 24	
Malic acid	malate dehydrogenase (Cla97C07G136660, Cla97C06G116670, Cla97C01G017230, Cla97C06G125760)	(Gong et al., 2021b)
	phosphoenolpyruvate carboxykinase (PEPCK)	(Yang et al., 2025)
	Cla97C03G054690 (malate synthase)	(Umer et al., 2020a)
	Cla97C06G125760 and Cla97C05G103110 malate dehydrogenases)	
	Cla008235 (malate dehydrogenases)	(Gao et al., 2018a)
	NAD-cyt MDH (malate dehydrogenases)	(Gao et al., 2018b)
	Cla97C02G049340 (transmembrane malate transport)	(Umer et al., 2020a)
	Cla97C04G076530 and Cla97C11G218660 (Pyruvate kinase)	
	ALMT7	
	Aluminium activated malate transporters (WMALMT-3, WMMDH-1, and WMMDH-2, WMALMT-4 and WMALMT-5)	(Umer et al., 2020b)
	ALMT13 and ALMT8	(Aslam et al., 2020)
	mal8-5	(Pingli et al., 2023)
Carotenoid	WMU38667 (phytoene synthase 1)	(Guo et al., 2011)
	WMU23817 (sucrose synthase)	
	WMU41454 (lycopene beta cyclase)	(Guo et al., 2011; Liu et al., 2013; Chai and Sun, 2025)
	*Cla97C02G03827*0 (red chlorophyll catabolite reductase)	(Nie et al., 2023)
	Cla97C01G008760 (phytoene synthase)	
	phytoene desaturases	
	One ζ-carotene isomerase	
	one ζ-carotene desaturase	
	β-carotene isomerases	
	lycopene cyclases	
	β-carotene hydroxylases	
	zeaxanthin epoxidases	
	violaxanthin de-epoxidases	
	CmAPRR2	(Oren et al., 2019)
	Lycopene β-cyclase gene	(Wu et al., 2019)
Lycopene	Cla005011 (lycopene β-cyclase)	(Wang et al., 2019)
	Cla005012	
	lycopene β-cyclase gene	(Wu et al., 2019)
Pectin	Cla004120, Cla009966, Cla006648	(Anees et al., 2021)
Hemicellulose	Cla012351, Cla004251, Cla004120, Cla009966, Cla006648, Cla007092, Cla004119, Cla018816	
	Cla012507 and Cla016033	(Sun et al., 2020)
Cellulose	Cla007092, Cla004119, Cla018816, Cla004120, Cla009966, Cla006648	(Anees et al., 2021)
Protopectin	Cla007092, Cla004119, Cla018816, Cla004120, Cla009966, Cla006648	
Firmness	Cla012499, Cla012500, Cla012502, and Cla012507	(Sun et al., 2020)
Cell wall	Cla006266 (pectinesterase), Cla006576 (Hydroxycinnamoyl transferase), Cla007259 (Pectinesterase inhibitor), Cla007803 (Pectinesterase family protein), and Cla002573 (pectate lyase)	

Natural allelic variations have also been reported for the observed diversity in watermelon pigmentation. In the coding region of lycopene β-cyclase (LCYB; Cla005011), two nonsense mutations, SNP1 (C/G) and SNP2 (A/C), revealed different colors of watermelon fruits. The varieties with the C allele have revealed increased β-carotene, and the ones with the G allele have shown increased β-apocarotenal production that leads to yellow- or orange-fleshed watermelon fruit (Pingli et al., 2023). On the other hand, in the SNP2 (A/C), the variety with the C allele has revealed more lycopene and has deciphered the red flesh color (Bang et al., 2007). Interestingly, these authors reported C_1_A_2_, G_1_A_2_, and C_1_C_2_ allele combinations of the above two SNPs that have been exhibited as pale, yellow, or orange and red or pink in wild, domesticated, and improved watermelon fruit, respectively. The development of molecular markers for PSY1 and lycopene β-cyclase (LCYB) has significantly advanced breeding efforts, enabling precise selection of flesh color traits to meet consumer preferences and enhance the marketability of new cultivars (Nie et al., 2023). As carotenoid is a polygenic trait, different genes have also been indicated as in the following **Table 2**.

### 3.4 Organic acid-related genes

Organic acids, such as malic acid, citric acid, and oxalic acid, contribute to the sourness and flavor complexity of watermelon fruit (Gao et al., 2018b; Umer et al., 2020b, Umer et al., 2020a). Their levels have been regulated by key genes, including phosphoenolpyruvate carboxylase (PEPC), malate dehydrogenase (MDH), citrate synthase (CS), and malate and citrate transporters, which have played essential roles in central carbon metabolism and organic acid transportation (**Table 2**). The genetic basis of organic acid content has been investigated through forward genetics, including transcriptomic, QTL, and GWAS approaches, and reverse genetics, including overexpression (Umer et al., 2020b) and CRISPR/Cas9 (Yang et al., 2025). During organic acid formation, several genes are involved in tricarboxylic acid (TCA) (**Figure 2B**). For example, comparative analysis of metabolomics and transcriptomics of watermelon cultivar “203Z” and its near-isogenic line “SW” has revealed the highly significantly expressed ALMT gene and its ortholog, Cla006064, the NAD-dependent malate dehydrogenase (NAD-cyt MDH) gene, and its ortholog, including Cla008235 and Cla011268, that have been positively correlated with malic acid accumulation (Gao et al., 2018b). These genes have synthesized acetyl CoA and malate or oxaloacetate. Similarly, these authors have reported upregulated citrate synthase (CS) that has manipulated citric acid contents of fruits. Transcriptomics and reverse transcription quantitative polymerase chain reaction (RT-qPCR) have also indicated high expression patterns of Cla97C07G128420 (ALMT), Cla97C03G068240 (CS), and Cla97C01G008870 (ICDH) genes that have been significantly correlated with malic acid and citric acid accumulations during key fruit developmental stages (Umer et al., 2020a). Further structural variation among different genotypes, validation, and utilization for molecular breeding are still the critical gaps that demand the engagement of researchers.

Gong et al. (2023) reported allelic variants that have been associated with reduced acidity. These discoveries are particularly valuable for breeding programs targeting markets that prefer milder-tasting fruit (Yang et al., 2025). By leveraging molecular markers linked to acid metabolism genes, breeders can more precisely fine-tune the flavor profiles of watermelon cultivars.

### 3.5 Amino acid-related genes

Watermelon is notable for its enormous amino acid content, including citrulline, arginine, glutamine, argininosuccinate, γ-aminobutyric acid, proline, valine, ornithine, phenylalanine, histidine, isoleucine, leucine, lysine, and alanine (Collins et al., 2007). Citrulline is a distinctive amino acid found in high concentrations in watermelon and has been recognized for its potential health benefits, including cardiovascular support and antioxidant activity (Burton-Freeman et al., 2021). Crimson Sweet watermelon has produced higher citrulline and arginine than “Dixielee” watermelon (Hartman et al., 2019). Citrulline content in watermelon shows moderate heritability, with estimates ranging from 38% to 43% in different populations, indicating a genetic basis for citrulline variation that can be exploited in breeding programs (Hartman, 2017). The genes ClCG05G018820 [ornithine carbamoyltransferase (OTC)] ClCG09G003180 [N-acetylornithine aminotransferase (N-AOA)], which have produced N-acetylornithine and N-acetylornithine deacetylase (AOD-3), have been involved in ornithine synthesis that has enhanced the accumulation of citrulline in watermelon fruit, while some genes involved in citrulline degradation include argininosuccinate synthases (ASS-1, ASS-2, and ASS-3), argininosuccinate lyases (ASL-1), ornithine decarboxylase (ODC), and arginine decarboxylase (ADC) to produce other amino acids (Joshi et al., 2019; Aslam et al., 2021). Some ornithine carbamoyltransferase genes, including Cla020511, Cla020512, and Cla020781 (ornithine carbamoyltransferase) have been candidate genes involved in the arginine accumulation (Fall et al., 2019). Moreover, the genes that have synthesized glutamate from 2-oxoglutarate of TCA to produce different amino acids are shown in **Figure 2B**. The genes that have been involved in different steps in the amino acid production pathway require further investigation to manipulate complete packages of amino acids. These discoveries facilitate the development of functional markers, enabling the breeding of nutrient-enriched watermelon varieties targeted at health-conscious consumers.

### 3.6 Volatile aromatic compound-related genes

The aroma of watermelon fruit is a crucial determinant of fruit quality, primarily governed by volatile organic compounds (VOCs) such as aldehydes, alcohols, esters, terpenes (Lu et al., 2024), apo-carotenoids, ketones, olefins, and furans (Bianchi et al., 2019). These compounds have been biosynthesized through fatty acid, amino acid, terpenoid, alcohol/aldehydes (ADH), ethyl acetate, acetaldehyde, tetradecanoic acid, and methyl acetate pathways regulated by key genes including lipoxygenases (LOX), alcohol dehydrogenases (ADH), and alcohol acyltransferases (AAT) (Gong et al., 2023). Watermelon genotypes have exhibited substantial diversity in their VOC profiles, resulting in distinct aromatic characteristics. Genes like lipoxygenases (LOX) (Liu et al., 2020), alcohol dehydrogenase genes (Cla97C01G013600, Cla97C05G089700, Cla97C01G001290, Cla97C05G095170, and Cla97C06G118330), and alcohol transferase genes (Gong et al., 2021a) have been involved in the metabolism of aromatic substances of watermelon fruit. There have been limited comparative transcriptome analyses involving wild and cultivated watermelon fruit to insight extensive variations in aroma development at various fruit developmental stages. While the genetic regulation of VOC biosynthesis remains complex, emerging research has identified some candidate genes linked to desirable aroma traits. These advances offer significant potential for targeted breeding strategies to improve flavor quality and sensory appeal in new cultivars.

### 3.7 Texture-related genes

Fruit texture is a critical quality trait that influences consumer acceptance and marketability of watermelon (Liu et al., 2024). Watermelon fruit texture is manipulated by cell wall components, including pectin, cellulose, and hemicellulose content, and identifying key genes involved in fruit texture regulation (Gao et al., 2020). Anees et al. (2021) identified key gene networks involving cell wall biosynthesis and the ethylene pathway (**Table 2**), providing insights for watermelon texture improvement in the future. Sun et al. (2020) also figured out multiple genes that have influenced watermelon fruit flesh firmness and consequently have affected cell wall components and hardness of ripened fruit (**Table 2**). Yang et al. (2023) reported that the Aux/IAA gene controls watermelon flesh firmness and influences fruit texture, with overexpression leading to increased flesh firmness and reduced ABA content. The Aux/IAA gene, Cla004102, has reduced flesh firmness of watermelon fruit when exposed to ethylene, and its expression has been decreased with fruit development stages (Anees et al., 2023). Sun et al. (2020) also reported fruit firmness controlling plant growth regulator genes, including Cla013991 (IAA-amido synthetase), Cla023158 (ethylene-responsive transcription factor), Cla016785 (ethylene-responsive transcription factor), Cla022055 (gibberellin receptor), Cla015407 (gibberellin 3-beta-hydroxylase), Cla005404 (9-cis-epoxycarotenoid dioxygenase), Cla016195 (ABA receptor), and Cla015981 (1-aminocyclopropane-1-carboxylate oxidase). These genes all need further validation and employment for fruit quality improvements through molecular breeding.

### 3.8 Flavonoids-related genes

Flavonoids in watermelon fruit contribute to its antioxidant properties, helping to protect cells from oxidative stress (Neglo et al., 2021). Gong et al. (2021b) reported more than 113 flavonoids that have been increased during key fruit developmental stages in wild watermelon “PI 632,751” and vice versa in cultivated watermelon “Cheng Lan” fruits. The flavonoid biosynthesis and accumulation have been regulated by genetic bases. Pingli et al. (2023) uncover the flavanone3-hydroxylase (Cla006682) gene, and Oren et al. (2019) figure out the CmAPRR2 gene that has been involved in flavonoid metabolism in watermelon fruit. Similarly, four genes, which are flavonoid 3 ′-monooxygenase (CYP75B1), phenolic glucoside malonyltransferase (PMAT1), isoflavone 2 ′-hydroxylase (CYP81E), and vestitone reductase (VR), have been deciphered (Peng et al., 2025). Flavonoids like luteolin and quercetin have been synthesized by the cytochrome P450 monooxygenase family gene, particularly CYP75B1 (Cla97C06G125240), in the flavonoid and flavonol pathways. Some of the unlocked genes have been involved in the phenylpropanoid pathway to regulate flavonoid accumulation in watermelon, as shown in **Figure 2C**. As it is one of the polygenic traits, further gene discovery needs to be employed and followed by validation and modern breeding approaches.

## 4 Applications in watermelon breeding

### 4.1 Marker-assisted selection

Marker-assisted selection (MAS) utilizes DNA markers linked to target traits to enhance breeding efficiency (Wang et al., 2019). The integrated genetic maps using SNPs, SSRs, insertion–deletion (InDels), and other markers have facilitated the identification and selection of desirable alleles for watermelon fruit metabolome improvements that have contributed to quality traits (Ren et al., 2014; Li et al., 2018). In watermelon fruit metabolite breeding programs, MAS has proven particularly effective for several key traits like sugar (using markers for *INV* and *SPS* genes), flesh color (via *LCYB* markers) (Wang et al., 2019), and citrulline content (through *ASS1* markers) (Mashilo et al., 2022). This approach has offered significant advantages over conventional methods by enabling early selection of superior genotypes, thereby reducing both the time and costs associated with traditional phenotypic screening while simultaneously improving breeding accuracy. This method should also be employed for other genes to select genotype and improve the fruit metabolite profile.

### 4.2 Genomic selection

Genomic analysis deciphered the evolutionary history of watermelon fruit metabolites through identifying the gene/s that manipulate sweetness, bitterness, sourness, color, and texture during domestication and improvement, providing targets for genome selection (Guo et al., 2019; Katuuramu et al., 2023). Genomic selection (GS) utilizes genome-wide molecular markers and statistical prediction models to calculate breeding values (Wu et al., 2019). Genome-wide association analysis (GWAS) studies have identified QTL and SNP markers associated with soluble fruit metabolites that impact flesh color, flavor, and aroma to reveal phenotypic variation (Wu et al., 2019). When incorporated with metabolomic data, GS achieves greater predictive accuracy, particularly for complex polygenic traits such as flavor compounds and aroma profiles (Guo et al., 2012, Guo et al., 2019). This integrated approach empowers researchers to make data-driven selection decisions by combining genomic and metabolic insights, which significantly accelerates the development of superior cultivars, though limited validation studies exist for genomic prediction accuracy. The narrow genetic basis of watermelon also limits the application of gene discovery for genome selection (Wu et al., 2019).

### 4.3 Gene editing and precision breeding

**Figure 3:**
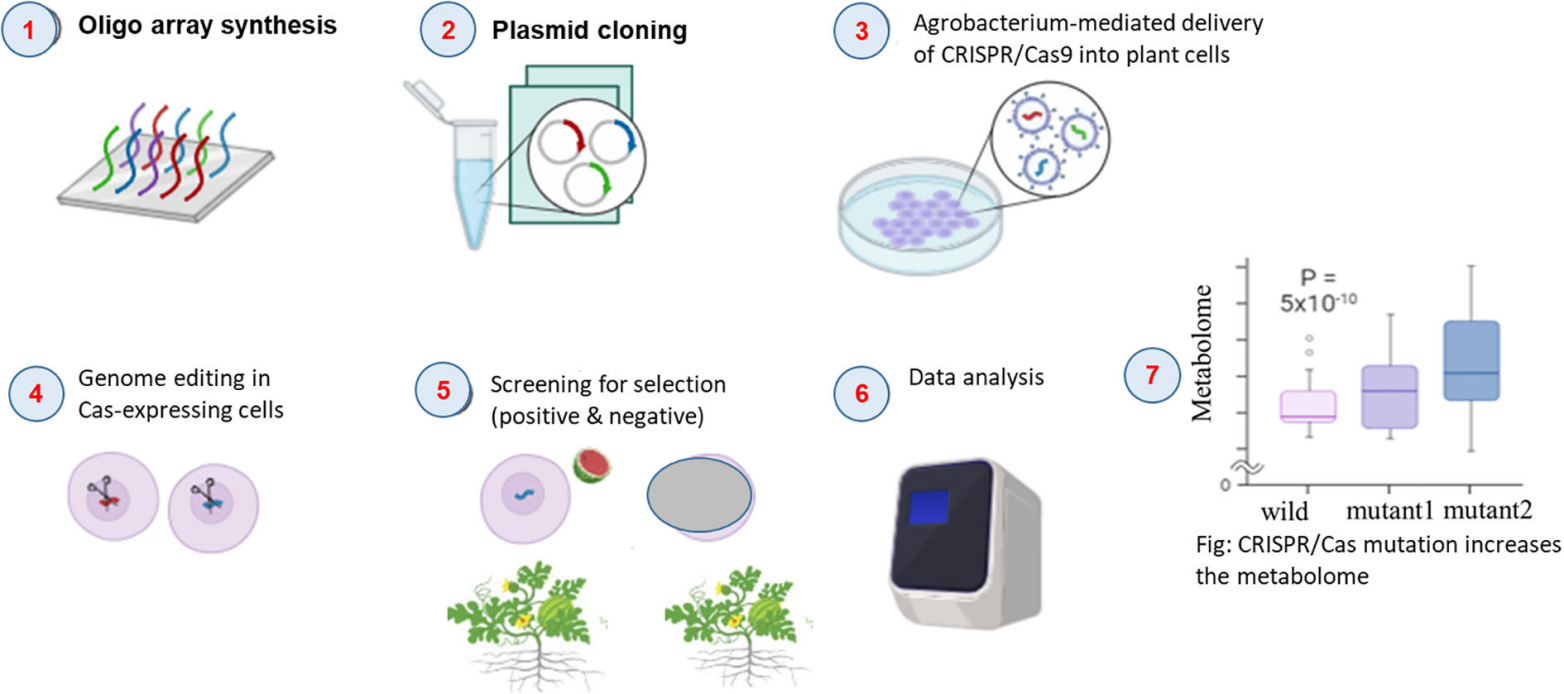
Model steps of CRISPR/Cas9 gene editing approach.

Recent advances in gene editing technologies, particularly clustered regularly interspaced short palindromic repeats-associated protein 9 (CRISPR/Cas9) systems, have commenced in the transformation of precision breeding in watermelon by enabling targeted modifications of key metabolic genes (Wang et al., 2024). Unlike conventional transgenic methods, gene editing provides a non-GMO strategy for trait improvement, making it particularly valuable for markets with strict biotechnology regulations (Lemmon et al., 2018). The technique’s precision in editing specific nucleotide sequences without introducing foreign DNA addresses major consumer concerns about genetically modified organisms (Tsakirpaloglou et al., 2023). As regulatory frameworks worldwide increasingly recognize the distinction between gene-edited and transgenic crops, these technologies are emerging as powerful tools for developing improved watermelon metabolites. Knock-in, knockdown, and knockout of genes are three distinct CRISPR/Cas9 mechanisms to alter the function of the genes. The model of general steps is indicated in **Figure 3**. Even though knockout has been emerging, it has been prominently featured in watermelon fruit metabolome research when compared with knockdown and knockin, which has not yet been employed to manipulate these traits. For instance, knockout of phosphoenolpyruvate carboxykinase (PEPCK), an organic acid-synthesizing gene, has reduced the acidity of watermelon fruits by hindering the function of this gene (Yang et al., 2025). The ClIAA16 mutant, which has been developed through knockout, has exhibited premature termination of protein translation due to one base pair insertion, which has shown delayed watermelon fruit ripening. This mutation may contribute to extended shelf life, though it has reduced the fruit sugar content (Hu et al., 2023). Similarly, two ClVST1 mutants of the ZZJM watermelon cultivar, with 5 and 1 nucleotide deletions, have been developed through the knockout approach and have revealed reduced sugar contents (Ren et al., 2021). These examples indicate promising potential of this breakthrough approach, which allows precise regulation of gene function to reduce undesirable traits through knockout and to enhance desirable characteristics such as sweetness and flesh color through knock-in of sugar metabolism genes (e.g., SPS, SuSy, fructokinase) and modulation of pigmentation via lycopene biosynthesis genes such as ClPSY1 in watermelon fruit. By combining gene editing with traditional breeding methods, researchers can accelerate the creation of varieties with optimized metabolomes that can improve fruit metabolite profiles and enhance nutritional contents, positioning this technology as a cornerstone of future watermelon improvement programs. Still, this approach is very infantile and requires more attention for the improvement of watermelon fruit metabolites that pertain to fruit quality.

## 5 Challenges and future perspectives

### 5.1 Challenges and limitations

While substantial advances have been made, several key challenges persist in watermelon metabolome gene discovery and its application in breeding programs. Capturing a diverse and dynamic range of thousands of compounds requires multiple analytical platforms such as GC-MS, LC-MS, NMR, refractometers, and penetrometers, which are labor intensive, destructive, costly, and complex. Each metabolite or group of metabolites requires different sample preparation and measurement. The reproducibility and quantitative accuracy have still been hard to guarantee for comparative studies, as inconsistent annotation has been revealed. Differences in metabolite composition and their accumulation between tissues at different developmental stages have been reported in watermelon (Neglo et al., 2021), which demands layers of complex experiments to figure out causal genes. Furthermore, although thousands of metabolites have been characterized, only a few genes have been reported for some metabolites. Hence, the discovery of their pathways and genes underlying the enormous number of metabolites requires greater emphasis to exploit and utilize for breeding.

Uncovering genes of polygenic metabolites and utilizing them for breeding necessitates large breeding populations, sophisticated analytical models, and a number of validations for effective gene discovery and selection. Additionally, pinpointing the exact candidate gene of polygenic traits within QTL has been like finding a needle in a haystack. The combinations of these difficulties with the limited genetic diversity of elite watermelon cultivars have hindered the opportunities for novel gene and allele discovery (Levi et al., 2001; Kantor and Levi, 2018). However, candidate genes for some metabolites have been identified; their functional validation and utilization in molecular breeding, including MAS, genome selection, and gene editing, has remained limited due to inadequate infrastructure and skilled human resources.

The environmental sensitivity of metabolite expression, which often obscures genetic signals and complicates data interpretation, has been the other obstacle (He et al., 2022; Zhu et al., 2021; Chen et al., 2024). For instance, the ClPSY (Phytoene Synthase) gene, which is crucial for carotenoid biosynthesis, has been regulated by light and hormones to influence β-carotene accumulation (Wu et al., 2020). This makes it challenging to figure out and utilize such genes across multiple environments. The limited investigation of different genotypes with diverse metabolite background, due to limited germplasm, has also constrained efforts to robustly find enormous metabolite pathways and causal genes.

The lack of integration of genomics, transcriptomics, proteomics, and metabolomics data streams for comprehensive molecular mechanism insights of the watermelon fruit metabolome remains mysterious. On the other hand, the absence of AI-powered multi-omics investigation slowed progress in unlocking, validating, and utilizing target genes. Investigations into predicting gene expression and metabolite accumulation using remote sensing, based on climate and growth performance, are also lacking. Such approaches, combined with the early manipulation of gene expression to achieve desired metabolite levels, remain underexplored.

### 5.2 Future perspectives to accelerate gene discovery and breeding impact

Systematic integration of multi-omics is crucial to reveal gene-to-metabolite networks to provide a holistic view of how genetic information translates into the metabolic profile of fruits. To achieve this, several strategies are being pursued, including the implementation of high-throughput phenotyping platforms to capture precise trait measurements, collection and expansion of genetic resources through the characterization of wild relatives and cultivars (Guo et al., 2019; Wu et al., 2019), and the application of artificial intelligence (AI) to decipher complex genotype–phenotype relationships (Frey, 2018; Wu and Xie, 2024).

The development of AI-driven approaches to predict metabolomes and their molecular mechanisms, as well as the use of gene editing to enhance metabolite production without relying on labor-intensive, expensive, and complex wet-laboratory activities, will help overcome current limitations in gene discovery and advance molecular breeding strategies. The emerged x-ray imaging driven by AI that has been employed to classify seeds into viable seeds, nonviable seeds, and abnormal viable seeds demonstrates the potential of such technologies for innovation in fruit metabolome gene discovery. Moreover, establishing AI agents and integrating them into the multi-omics research pipelines, along with promoting partnerships and sharing data in digital approaches, can maximize the benefits of collaborative efforts. These integrated approaches hold the potential to accelerate the development of next-generation watermelon varieties with multiple enhanced metabolites. By digging out candidate genes and utilizing them for molecular breeding, researchers can generate cultivars with improved metabolome profiles and superior quality characteristics to fulfill evolving market demands.

## 6 Conclusions

The exploration of the watermelon fruit metabolome has entered a transformative era driven by multi-omics technologies, which have been instrumental in uncovering the genetic underpinnings of key metabolites such as sugar, cucurbitacin, carotenoids, volatile compounds, amino acids, organic acids, and flavonoids. The identified genes regulating sugars (SPS, INVs, SUS, *ClTST2*, *ClSWEET3*), cucurbitacin (OSC, ACT, CYP), carotenoids (*LCYB*, *PSY1*, CHYB), amino acids (OTC, N-AOA, AOD-3), organic acids (*PEPCK*, ALMT, ICDH, *CS*), and flavonoids (CmAPRR2, PMAT1, CYP81E, VR) provide a critical genetic background for modern breeding programs. The forward and reverse gene harvesting approach has revolutionized the sector. The application of these discovered genes through MAS, GS, and CRISPR/Cas9-mediated gene editing enables breeding to enhance desirable metabolites and suppress undesirable ones, offering an efficient pathway to develop superior cultivars. However, several significant challenges persist, including the polygenic and environmentally sensitive nature of metabolic traits, the limited genetic diversity of elite germplasms, the high cost and complexity of metabolomic phenotyping, and the limited application of molecular breeding. Future advancements hinge on systematically integrating multi-omics data, leveraging wild genetic resources to broaden diversity, and employing AI to decipher complex gene-to-metabolite networks and predict genetic bases and phenotypic outcomes, thereby accelerating the development of next-generation watermelon varieties with optimized fruit metabolites to meet evolving consumer and market demands.
